# PREVALENCE AND PREDICTORS OF STATIN USE IN LONG-STAY NURSING HOME RESIDENTS WITH LIFE-LIMITING ILLNESSES

**DOI:** 10.1093/geroni/igz038.3164

**Published:** 2019-11-08

**Authors:** Deborah S Mack, Kate L Lapane

**Affiliations:** 1 University of Massachusetts Medical School, Graduate School of Biomedical Sciences, Department of Population and Quantitative Health Sciences, Worcester, Massachusetts, United States; 2 University of Massachusetts Medical School, Department of Population and Quantitative Health Sciences, Worcester, Massachusetts, United States

## Abstract

Statins are one of the most commonly prescribed medications in the United States. While statin use has been studied extensively in the general population, national data on statin use in US nursing homes do not exist. This study estimated the point prevalence of statin use on September 1, 2016 and identified predictors of statin use in nursing home residents with life limiting illness. We conducted a cross-sectional analysis using national MDS 3.0 data linked to Medicare claims. We identified 424,312 long-stay residents with life limiting illnesses defined as a palliative care consultation (ICD-10 Z51.5), prognosis <6 months on MDS, the Veterans Health Administration palliative care index (PCI), or a diagnosis of a serious illness (e.g., cancer, stroke, heart failure, etc.). Poisson models accounted for clustering of residents within facilities. Overall, 34% were on statins which varied by age (65-75 years: 44.1%; >75 years: 31.5%). The strongest positive predictor of statin use was hyperlipidemia, while coronary artery disease and stroke were only marginally predictive across age. The strongest negative predictors were a palliative care consultation or a prognosis <6 months, while PCI was not strongly associated with use. A substantial proportion of long stay nursing home residents with life limiting illnesses continue statin therapy despite evidence of net harm. Efforts to deprescribe statins in the nursing home setting may be warranted. These findings can be used to help identify and target missed opportunities to reduce the therapeutic burden and improve end-of-life care for the US nursing home population.

